# Calcium Dobesilate (CaD) Attenuates High Glucose and High Lipid-Induced Impairment of Sarcoplasmic Reticulum Calcium Handling in Cardiomyocytes

**DOI:** 10.3389/fcvm.2021.637021

**Published:** 2021-02-02

**Authors:** Jianxin Deng, Xiangsheng Cai, Mingyu Hao, Xueting Liu, Zelong Chen, Haiyan Li, Junying Liu, Yunxiu Liao, Hao Fu, Huiyan Chen, Gangjian Qin, Dewen Yan

**Affiliations:** ^1^Department of Endocrinology, Shenzhen Second People's Hospital, The First Affiliated Hospital of Shenzhen University, Health Science Center of Shenzhen University, Shenzhen, China; ^2^Center for Medical Experiments, University of Chinese Academy of Science-Shenzhen Hospital, Shenzhen, China; ^3^School of Basic Medical Science, Health Science Center of Shenzhen University, Shenzhen, China; ^4^Molecular Cardiology Program, Department of Biomedical Engineering, University of Alabama at Birmingham, Birmingham, AL, United States

**Keywords:** calcium dobesilate, cardiomyocytes, calcium sparks, hyperglycemia, laser scanning confocal microscope

## Abstract

Calcium dobesilate (CaD) is used effectively in patients with diabetic microvascular disorder, retinopathy, and nephropathy. Here we sought to determine whether it has an effect on cardiomyocytes calcium mishandling that is characteristic of diabetic cardiomyopathy. Cardiomyocytes were sterile isolated and cultured from 1 to 3 days neonatal rats and treated with vehicle (Control), 25 mM glucose+300 μM Palmitic acid (HG+PA), 100 μM CaD (CaD), or HG+PA+CaD to test the effects on calcium signaling (Ca^2+^ sparks, transients, and SR loads) and reactive oxygen species (ROS) production by confocal imaging. Compared to Control, HG+PA treatment significantly reduced field stimulation-induced calcium transient amplitudes (2.22 ± 0.19 vs. 3.56 ± 0.21, *p* < 0.01) and the levels of caffeine-induced calcium transients (3.19 ± 0.14 vs. 3.72 ± 0.15, *p* < 0.01), however significantly increased spontaneous Ca^2+^ sparks firing levels in single cardiomyocytes (spontaneous frequency 2.65 ± 0.23 vs. 1.72 ± 0.12, *p* < 0.01) and ROS production (67.12 ± 4.4 vs. 47.65 ± 2.12, *p* < 0.05), which suggest that HG+PA treatment increases the Spontaneity Ca^2+^ spark frequency, and then induced partial reduction of SR Ca^2+^ content and subsequently weaken systolic Ca^2+^ transient in cardiomyocyte. Remarkably, these impairments in calcium signaling and ROS production were largely prevented by pre-treatment of the cells with CaD. Therefore, CaD may contribute to a good protective effect on patients with calcium mishandling and contractile dysfunction in cardiomyocytes associated with diabetic cardiomyopathy.

## Introduction

The worldwide prevalence of diabetes mellitus increases markedly to cause various cardiovascular complications, which are the major cause of morbidity and mortality in this population (1, 2). Among patients with the same degree of cardiovascular diseases, the mortality rate from cardiovascular diseases in diabetic patients is 2–4 times higher than that of non-diabetic patients. In addition, diabetic cardiomyopathy can occur without any vascular pathogenesis (3–5); The disease progress from initial asymptomatic left ventricular (LV) diastolic dysfunction to impaired LV systolic function, and eventually advances to heart failure (6). Therefore, a better understanding of mechanisms of diabetes-associated cardiomyocytes impairments will aid the development of novel strategies for protecting heart function in these patients (7–9).

As we know, Ca^2+^ plays an essential role in cardiac EC-coupling. Ca^2+^ major stores in sarcoplasmic reticulum (SR) in the mammalian heart. At systole, instantaneous high concentration of Ca^2+^ is released from SR trigged by Ca^2+^ influx trough L-type Ca^2+^ channel, consequently Ca^2+^ transient that triggers myofilaments contraction in heart (10). Ryanodine receptors play essential role in Ca^2+^ release from SR *via* CICR mechanism. Spontaneous Ca^2+^ sparks, the elementary intracellular Ca^2+^ release from 4 to 6 neighboring RyRs, is sporadic occurs in the cadiomyocytes under physiological condition. The frequency of Ca^2+^ sparks is closely related to the activity of RyR at diastole, and control SR Ca^2+^ content. High Spontaneous Ca^2+^ sparks frequency, often happened in kind of diseases, have been reported to reduce SR Ca^2+^ content (11). RyR dysfunction has been shown to cause various of cardiac dysfunction, such as heart failure, hypertrophy (12, 13) and ischemia-reperfusion (14). Abnormal RyR function has also been shown in type 1 diabetes (15), and STZ-induced type-2 diabetes rat model (2), despite the pathological significance is not well-characterized.

Calcium dobesilate (CaD) is a vascular protective agent, highly effective in treating patients with various diabetes-associated microvascular disorders, retinopathy, nephropathy, and cataract (16–18). CaD has been shown to decrease intracellular ROS production, improve the biosynthesis of collagen in the basement membrane, and inhibit the high permeability caused by vasoactive substances. However, whether CaD has an effect on diabetes-associated or high glucose-induced cardiomyocytes dysfunction is currently unknown. In this study, we sought to examine the potential effects of CaD on calcium regulation of primary ventricular cardiomyocytes. We found that CaD efficiently protects high glucose-induced SR calcium leaks and depletion. Thus, CaD may be used to protect impairment of cardiomyocytes calcium signaling and contractile function associated with diabetic cardiomyopathy.

## Methods

### Isolation, Culture, and Treatments of Neonatal Rat Ventricular Myocytes (NRVMs)

Sprague–Dawley rats of 1–3-days-old were purchased from the Animal center of Guangdong Experimental Animal Center and handled according to a protocol approved by the Institutional Care and Use Committee of Shenzhen University, which conforms to the Guide for the Care and Use of Laboratory Animals published by the US National Institutes of Health (NIH Publication No. 85-23, revised 1996). NRVMs were isolated and cultured as previously described (19). After cultured for 24 h, the cells were stimulated with 25 mM glucose+300 μM PA (sigma, USA) for 24 h. In the case of CaD (Merck Serono Co., Ltd) treatment, NRVMs were pretreated with 100 μM CaD for 24 h.

### Ca^2+^ Spark Recording

Briefly, NRVMs loaded with Fluo-4 AM (ThermoFisher Scientific, F14201, dissolved in DMSO contains 20% pluronic F-127) for 10 min at 37°C and then perfused with Tyrode's solution (137 mM NaCl, 1.2 mM MgCl_2_, 1.2 mM NaH_2_PO_4_, 20 mM HEPES, 5.4 mM KCl, 1.8 mM CaCl_2_, 10 mM glucose, pH 7.4) in a recording chamber. Confocal line-scan imaging was carried out in resting cells at 488 nm excitation and 505 nm collection with a Zeiss 780 inverted confocal microscope (Carl Zeiss) with a 40x oil immersion lens for detecting Ca^2+^ sparks as described previously (20). Line-scan images were acquired at a sampling rate of 3.84 ms per line.

### Measurement of Action Potential (AP)-Elicited Ca^2+^ Transient

After cells loaded with Fluo-4 AM, Cells were superfused with a normal external solution containing (same as calcium spark recording) remove the dye and complete de-esterification. After the cardiomyocytes reached a steady-state with field stimulation (1 Hz), line-scan imaging was acquired along the longitudinal axis of the cells.

### Measurement of SR Ca^2+^ Content

Short puffs of caffeine (20 mM) were applied to completely empty the SR, after a train of 1-Hz field stimulation to achieve steady-state SR Ca^2+^ loading. SR Ca^2+^ content was assessed by detecting the amplitude of the Ca^2+^ transient. Cells were superfused with normal external solution.

### Measurement of Reactive Oxygen Species (ROS)

Cells were loaded with the probe 5-(6)-chloromethyl-2′,7′-dichlorodihydrofluorescein diacetate (CM-H2DCFDA, 10 μM, Molecular Probes) for 10 min at 37°C before the experiment. The 2′,7′-dichlorodihydrofluorescein (DCF) fluorescent intensity was measured by using LSM 780 (Zeiss) at an excitation wavelength of 485 nm and an emission wavelength of 530 nm (21).

### Statistical Analysis

All values are given as mean ± SE. Unpaired Student's *t*-test between 2 groups was performed, one-way analysis of variance (ANOVA) followed by the Dunnett's *post-hoc* test was used for comparisons among four groups. In all tests, values of *p* < 0.05 were considered be statistically significant.

## Results

### CaD Attenuates HG+PA–Induced Increase in Ca^2+^ Spark Frequency

**Figure 1:**
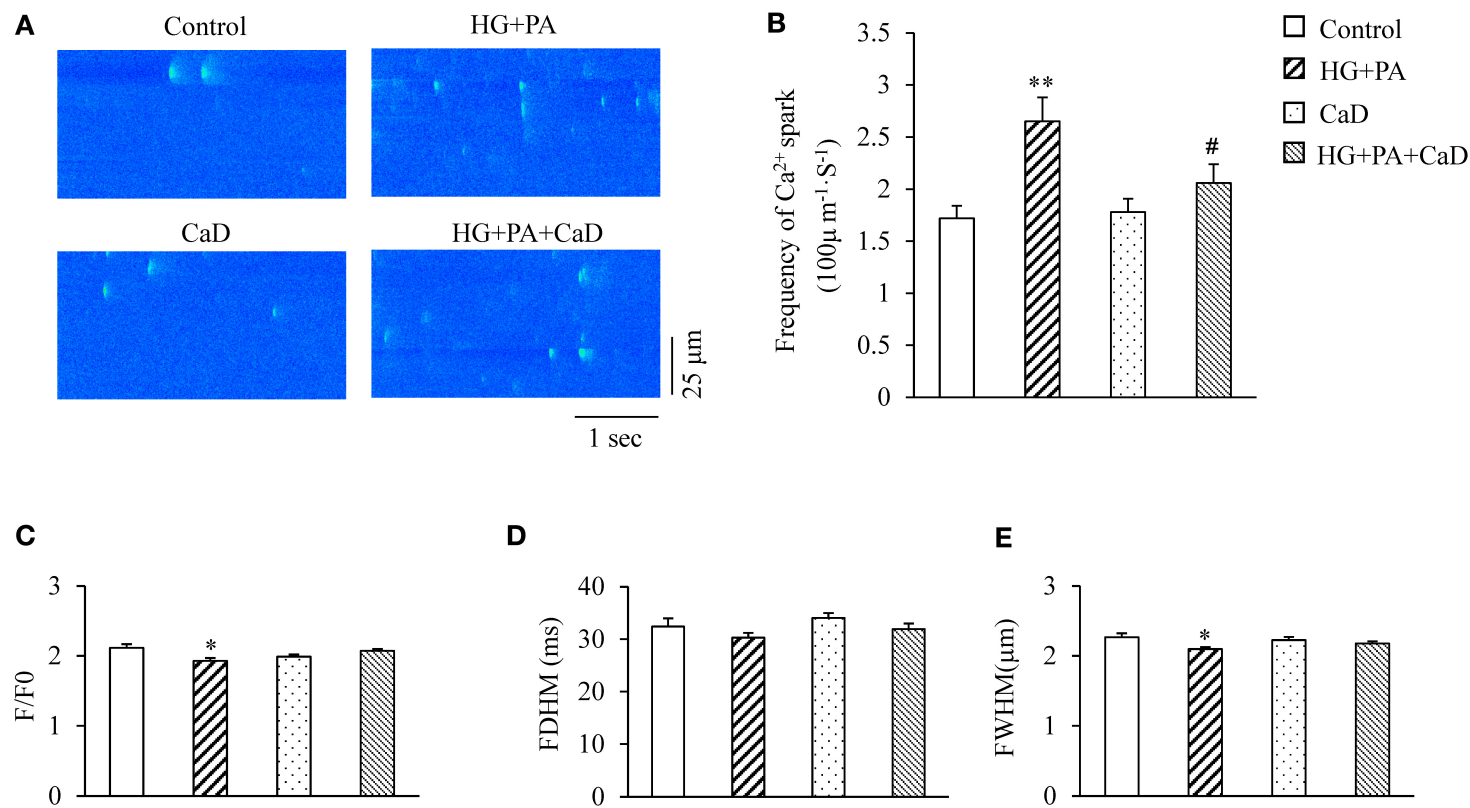
HG+PA increases Ca^2+^ sparks frequency in cardiomyocytes. **(A)** Representative Ca^2+^ spark images. **(B–E)** Statistic analyses of the frequency **(B)**, amplitude (F/F0, **C**), full duration of half maximum (FDHM, **D**) and full width of half maximum (FWHM, **E**) of Ca^2+^ sparks in Control, HG+PA, CaD, and HG+PA+CaD treatments. **p < 0.01, *p < 0.05 vs. control; ^#^*p* < 0.05 vs. HG+PA. *n* = 103–258.

We initiated our study by analyzing effects of HG+PA and CaD on the occurrence and properties of Ca^2+^ sparks in NRVMs. HG+PA increased the frequency of Ca^2+^ sparks by 1.54-fold (2.65 ± 0.23 vs. 1.72 ± 0.12 sparks/100 μm·s in Control, *p* < 0.01. Figures 1A,B) and to a lesser extent, decreased the amplitude(F/F0) of Ca^2+^ sparks (1.93 ± 0.04 vs. 2.12 ± 0.05 in Control, *p* < 0.05) (Figure 1C). In addition, HG+PA decreased full width of half maximum (FWHM) of Ca^2+^ sparks but did not alter the full duration of half maximum (FDHM) (Figures 1D,E). Remarkably, pre-treatment of cells with CaD restored HG+PA–induced alterations in Ca^2+^ spark frequency, amplitudes, and FWHM (Figures 1A–E). These results suggest that CaD normalizes HG+PA induced RyR dysfunction in the cardiomyocytes at baseline.

### CaD Attenuates HG+PA–Induced Reduction in SR Ca^2+^ Content

**Figure 2:**
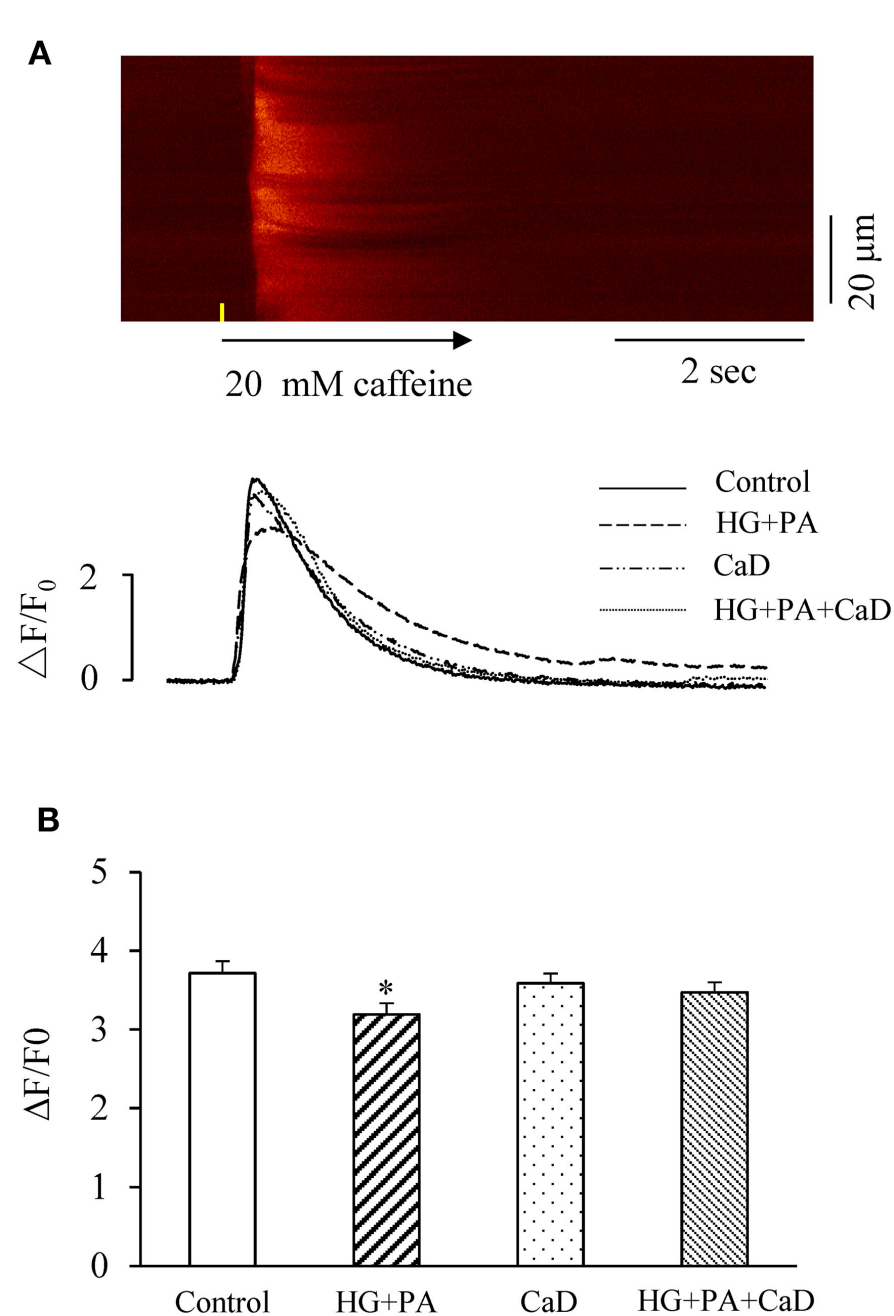
HG+PA reduces SR Ca^2+^ content in cardiomyocytes. **(A)** Representative confocal image in control and representative time-courses of caffeine-elicited Ca^2+^ transient traces in Control, HG+PA, CaD, and HG+PA+CaD. **(B)** Average SR Ca^2+^ contents indicated by the amplitude of caffeine-elicited Ca^2+^ transient (ΔF/F0) in Control, HG+PA, CaD, and HG+PA+CaD. *p < 0.05 vs. control. *n* = 10–20.

SR Ca^2+^ content affects the frequency and amplitude of Ca^2+^ sparks in cardiomyocytes; the higher of SR Ca^2+^ content results in the higher frequency of spontaneous Ca^2+^sparks (22). To determine whether HG+PA–increased increase in the spark frequency is due to an increased SR load, we analyzed the amplitude of caffeine-elicited Ca^2+^ transient (ΔF/F0) indicating SR Ca^2+^ content. High concentration of Caffeine (20 mM) was used to stimulate the change of calcium storage capacity in each group. We found that HG+PA treatment significantly decreased SR Ca^2+^ content (ΔF/F0, Figure 2A). In contrast, pre-treatment of cells with CaD completely prevented the HG+PA–induction of SR Ca^2+^ depletion (Figure 2B).

### CaD Ameliorates HG+PA–Induced Reduction in Systolic Ca^2+^ Transient

**Figure 3:**
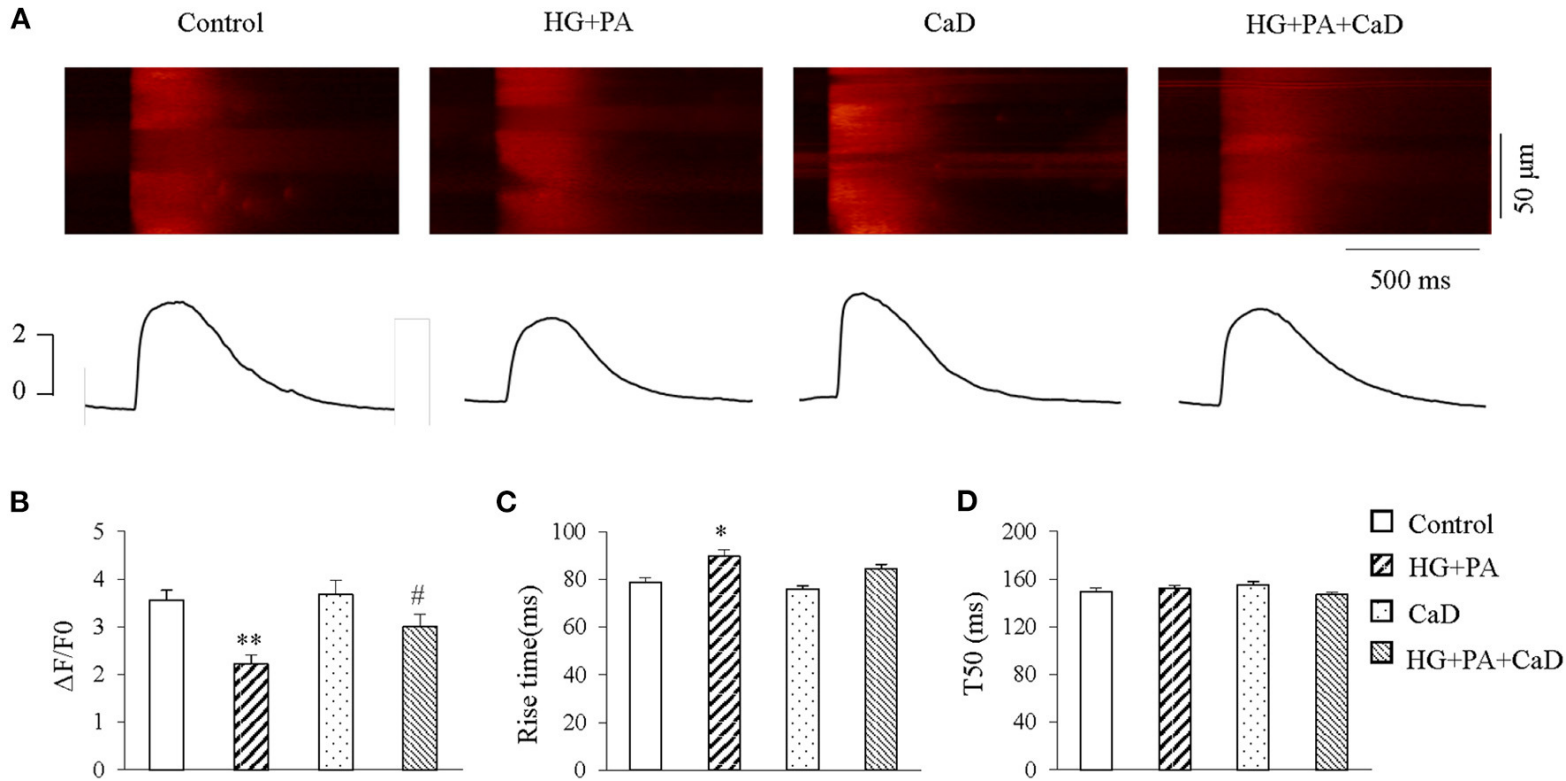
HG+PA reduces systolic Ca^2+^ transient in cardiomyocytes. **(A)** Representative confocal line-scan images of Ca^2+^ transients (*upper panels*) along with the spatial average (*lower panels*) in Control, HG+PA, CaD, and HG+PA+CaD treatments. **(B–D)** Average amplitudes (ΔF/F0, **B**), Rise time **(C)** and half time of decay (T_50_, **D**) of Ca^2+^ transient in Control, HG+PA, CaD, and HG+PA+CaD treatments. **p < 0.01, *p < 0.05 vs. control; ^#^*p* < 0.05 vs. HG+PA. *n* = 25–45.

Previous reports suggest that the reduction of systolic calcium transients in cardiomyocytes is the main cause of impaired contractility in diabetic cardiomyopathy (15). We elicited systolic Ca^2+^ transient by 1 Hz-field stimulation in the treatment groups. HG+PA treatment caused a significantly reduced amplitude (ΔF/F0, 2.22 ± 0.19 vs. 3.56 ± 0.21 in Control, *p* < 0.01, Figures 3A,B) and a significantly prolonged rise time (Figure 3C) of Ca^2+^ transient, suggesting an impairment of the synchrony of SR Ca^2+^ release. Treatment with CaD did not affect the calcium transient amplitude (3.68 ± 0.3 vs. 3.56 ± 0.21 in Control, *p* > 0.05), however, ameliorated HG+PA–induced impairment of SR Ca^2+^ release (ΔF/F0, 3.28 ± 0.25 vs. 2.22 ± 0.19 in HG+PA, *p* < 0.05; Rise time, 89.68 ± 2.81 vs. 78.59 ± 2.12 in HG+PA, *p* < 0.05. Figures 3A–C). We did not observe a statistical significance in T50 between the four treatment groups (Figure 3D).

### CaD Suppress HG+PA–Induced Oxidative Stress

**Figure 4:**
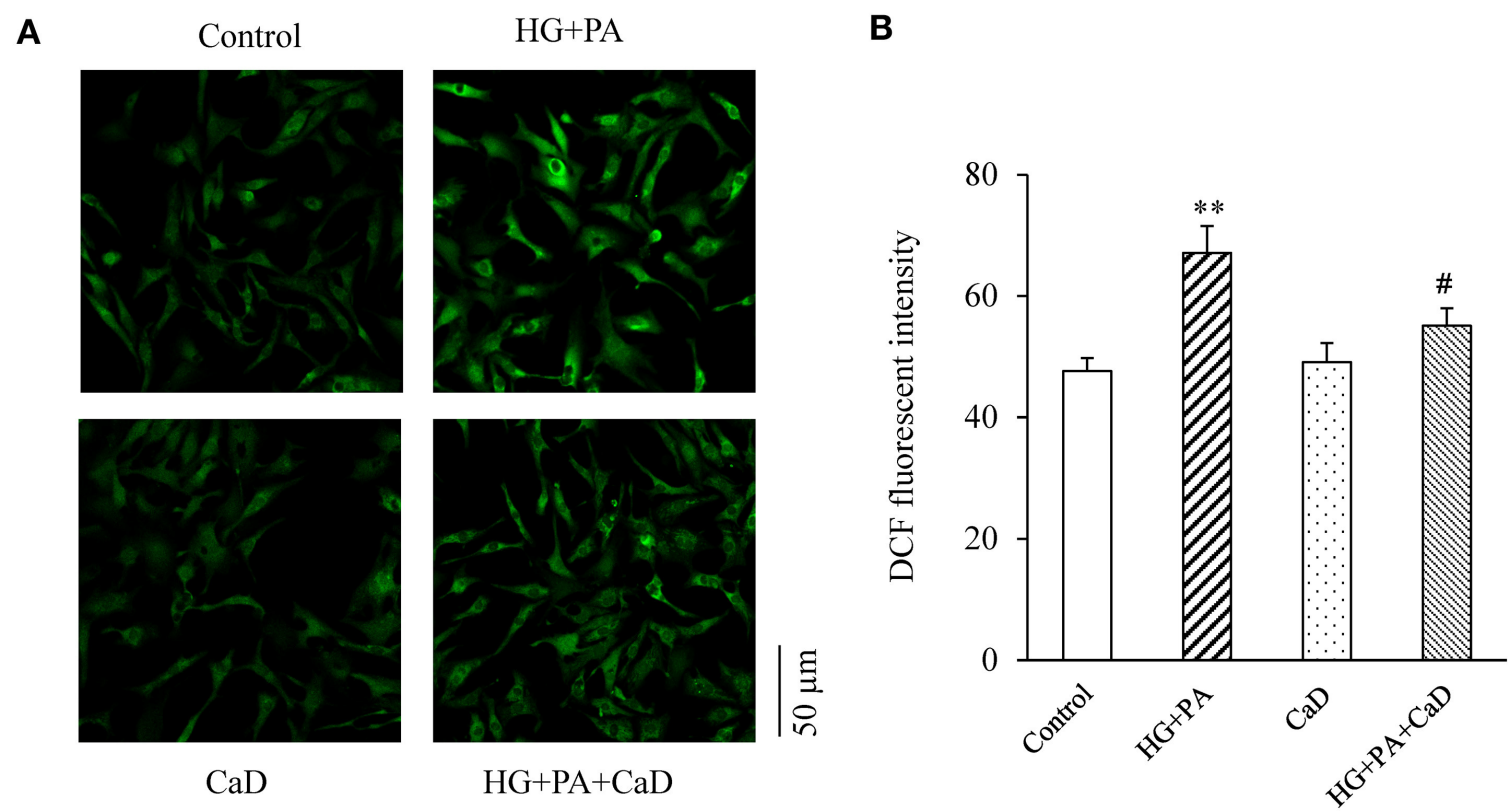
HG+PA increases the level of ROS in neonatal cardiomyocytes. Representative fluorescence images **(A)** and quantification **(B)** of ROS production. **p < 0.01 vs. control; ^#^*p* < 0.05 vs. HG+PA.

Because oxidation and nitrosylation of cysteine residues on RyR are key post-translational modifications that make the channel open to Ca^2+^ leaks, whereas reversal of these modifications closes the channel (23, 24), we examined whether the HG+PA induced hyperactive Ca^2+^ sparks is accounted by the increased ROS level in cardiomyocytes. We found that HG+PA treatment dramatically increased superoxide concentration in cardiomyocytes, and this change was markedly attenuated by treatment with CaD (Figure 4). Thus, CaD suppresses HG+PA–induced ROS production.

## Discussion

In this study, we found that exposure of neonatal rat ventricular cardiomyocytes to high glucose and high lipid (HG+PA) results in a marked increase in the intracellular ROS and Ca^2+^ sparks the frequency, a partial depletion of the SR Ca^2+^ content, and contractile dysfunction. Treatment of these cells with CaD attenuates the ROS production, spontaneous Ca^2+^ sparks, and SR Ca^2+^ depletion, restoring cardiomyocyte function. Although the beneficial effects of CaD in diabetic microangiopathy (e.g., diabetic retinopathy, nephropathy, and neuropathy) have been well-known, we study for first-time documents that CaD protects cardiomyocytes from HG+PA induced injury and Ca^2+^ mishandling.

Our findings have a high translational value for the treatment of diabetic cardiomyopathy. Since the 1970's, CaD has been front-line medication for the prevention and treatment of diabetic retinopathy with an excellent safety profile. Our data suggest that it may have a direct protective effect on cardiomyocytes in diabetes and that its use may be extended to diabetic heart disease. This is particularly important because currently there is no drug to specifically target high-glucose and high-fat induced cardiomyocyte injury. Notably, our results indicate that CaD is effective after HG+PA treatment and does not affect cardiomyocyte function at baseline. Therefore, it is probably safe and consistent with its safety profile in the clinic.

Diabetes mellitus is a metabolic disorder characterized by abnormal glucose and lipid metabolism. Hyperglycemia and high fat can cause cardiac damage, leading to diabetic cardiomyopathy (3, 4, 25). Mechanistically, long-time high blood glucose leads to excessive protein glycosylation and accumulation of advanced glycation end products (AGEs), which in sync with an elevation of the receptor for AGEs (RAGE) in the HG milieu, induces the production of cytokines and free radicals, resulting in cardiomyocyte injury. Importantly, we have previously shown that superfluous AGEs can influence Ca^2+^ spark firing frequency in cardiomyocytes, and that inhibition of RAGE significantly reduces the calcium spark frequency and protects heart function (26). Additionally, high blood glucose may induce apoptosis of cardiomyocytes through the deposition of oxidative stress, and now ample evidence suggests an increase of RyR in diabetes that is associated with oxidative stress. In addition to hyperglycemia, high fat is also found to induce ROS production. For example, free fatty acid (FFA) can induce cardiomyocyte apoptosis by increasing the concentrations of ceramide and ROS. Because overproduction of ROS contributes to the pathogenesis of diabetic cardiomyopathy, treatment with antioxidants can reverse the cardiac dysfunction (27, 28).

Recently, a series of studies found that SR calcium leakage increases in the cardiomyocytes of heart failure patients and contributes to the pathogenesis of heart failure (29, 30). Notably, ROS has been shown directly associated with Ca^2+^ sparks in the cardiomyocytes (31, 32). Enhanced diastolic SR Ca^2+^ leak has been suggested to be a potential cause of reduction of SR Ca^2+^ content (12), and the systolic intracellular Ca^2+^ concentration determines cardiac contractility. In rodent heart, Ca^2+^ influx through LCC accounts for no more than 30% while SR Ca^2+^ release contributes to over 70% of Ca^2+^ transient in systolic (33). Our data suggested that HG+PA significantly decreased SR Ca^2+^ content, which is consistent with the alteration of the magnitude of the systolic Ca^2+^ transient. Our finding indicates that HG+PA decreases cardiac contractile function, which may be an important mechanism for cardiac dysfunction in diabetic cardiomyopathy.

CaD, as an oxygen free radical scavenger, have been shown inhibits free radical production both *in vitro* and *in vivo* (34–37). In this study, we found that HG+PA significantly increases the oxidative stress level in cardiomyocytes, and this effect was effectively attenuated by CaD treatment. Thus, CaD reduces oxidative stress levels in the cells, thereby reducing the frequency of spontaneous calcium sparks, restoring the sarcoplasmic reticulum calcium pool, and stabilizing calcium transients in cardiomyocytes.

One limitation of this study is that our experimental system was primarily based on the cultured neonatal rat cardiomyocytes, which may have electrophysiological features different from the adult cells or the *in vivo* diabetic cardiomyopathy models. Thus, cautions should be taken when translating these findings into potential clinic use of the drug. In addition, the molecular targets of CaD that mediates its effects on ROS reduction and consequent Ca^2+^ signaling in cardiomyocytes are yet to be identified, which remains a subject of our future studies.

In conclusion, CaD protects cardiomyocytes from HG and PA-induced elevation in oxidative stress and spontaneous spark frequency and restores sarcoplasmic reticulum calcium levels. Thus, it may be utilized for the treatment of cardiac contractile dysfunction in diabetic cardiomyopathy.

## Data Availability Statement

The raw data supporting the conclusions of this article will be made available by the authors, without undue reservation.

## Ethics Statement

The animal study was reviewed and approved by Laboratory Animal Science Research Committee of Shenzhen University Health Science Center.

## Author Contributions

JD and DY conceptualized the study and contributed to resources. XC, ZC, MH, YL, HF, HC, HL, and JL collected data. JD and GQ wrote the manuscript. All authors contributed to the article and approved the submitted version.

## Conflict of Interest

The authors declare that the research was conducted in the absence of any commercial or financial relationships that could be construed as a potential conflict of interest.
